# Action Opportunities to Pursue Responsible Digital Care for People With Intellectual Disabilities: Qualitative Study

**DOI:** 10.2196/48147

**Published:** 2024-02-28

**Authors:** Nienke M Siebelink, Kirstin N van Dam, Dirk R M Lukkien, Brigitte Boon, Merlijn Smits, Agnes van der Poel

**Affiliations:** 1 Academy Het Dorp Arnhem Netherlands; 2 Tranzo Tilburg School of Social and Behavioral Sciences Tilburg University Tilburg Netherlands; 3 Vilans Utrecht Netherlands; 4 Copernicus Institute of Sustainable Development Utrecht University Utrecht Netherlands; 5 Siza Arnhem Netherlands; 6 PBLQ The Hague Netherlands

**Keywords:** ethics, value-based health care, digital technology, intellectual disability, digital care

## Abstract

**Background:**

Responsible digital care refers to any intentional systematic effort designed to increase the likelihood of a digital care technology developed through ethical decision-making, being socially responsible and aligned with the values and well-being of those impacted by it.

**Objective:**

We aimed to present examples of action opportunities for (1) designing “technology”; (2) shaping the “context” of use; and (3) adjusting the behavior of “users” to guide responsible digital care for people with intellectual disabilities.

**Methods:**

Three cases were considered: (1) design of a web application to support the preparation of meals for groups of people with intellectual disabilities, (2) implementation of an app to help people with intellectual disabilities regulate their stress independently, and (3) implementation of a social robot to stimulate interaction and physical activity among people with intellectual disabilities. Overall, 26 stakeholders participated in 3 multistakeholder workshops (case 1: 10/26, 38%; case 2: 10/26, 38%; case 3: 6/26, 23%) based on the “guidance ethics approach.” We identified stakeholders’ values based on bottom-up exploration of experienced and expected effects of using the technology, and we formulated action opportunities for these values in the specific context of use. Qualitative data were analyzed thematically.

**Results:**

Overall, 232 effects, 33 values, and 156 action opportunities were collected. General and case-specific themes were identified. Important stakeholder values included quality of care, autonomy, efficiency, health, enjoyment, reliability, and privacy. Both positive and negative effects could underlie stakeholders’ values and influence the development of action opportunities. Action opportunities comprised the following: (1) technology: development of the technology (eg, user experience and customization), technology input (eg, recipes for meals, intervention options for reducing stress, and activities), and technology output (eg, storage and use of data); (2) context: guidelines, training and support, policy or agreements, and adjusting the physical environment in which the technology is used; and (3) users: integrating the technology into daily care practice, by diminishing (eg, “letting go” to increase the autonomy of people with intellectual disabilities), retaining (eg, face-to-face contact), and adding (eg, evaluation moments) certain behaviors of care professionals.

**Conclusions:**

This is the first study to provide insight into responsible digital care for people with intellectual disabilities by means of bottom-up exploration of action opportunities to take account of stakeholders’ values in designing technology, shaping the context of use, and adjusting the behavior of users. Although part of the findings may be generalized, case-specific insights and a complementary top-down approach (eg, predefined ethical frameworks) are essential. The findings represent a part of an ethical discourse that requires follow-up to meet the dynamism of stakeholders’ values and further develop and implement action opportunities to achieve socially desirable, ethically acceptable, and sustainable digital care that improves the lives of people with intellectual disabilities.

## Introduction

### Digital Care

As digital tools have shown great potential to enhance health care and well-being services, digital care plays a central role in the policies and plans of governments and care organizations to continue to provide good care efficiently [1-3]. Digital care refers to technology and data that inform and improve health care provision [4]. Unfortunately, today, digital care is often not aligned with the needs and values of its users and other stakeholders [5,6]. Not aligning digital care with stakeholders’ needs and values results in low technology uptake. Although the importance of involving all relevant stakeholders in digital care innovation is widely accepted [7,8], they are insufficiently involved and, often, involved very late in digital care innovation [9]. Consequently, time and effort are wasted [10], and the clinical appropriateness and usability of digital care are compromised [11].

Digital care is found to have a significant impact on people’s lives, especially for those with intellectual disabilities who often receive life-long care. For people with intellectual disabilities, technologies are not only applied to promote health but also to enhance independence and quality of life, such as being able to participate in the society, which creates more educational, vocational, and leisure opportunities [12-14]. In long-term care organizations, integrating technology in daily practice for people with intellectual disabilities requires insight into the needs and values of the people with intellectual disabilities themselves, as well as those of their care professionals who guide them through daily life and need to adapt their guiding strategies, the IT support staff of the care organization who provide technical support for the digital solutions, the human resources professionals who need to integrate the technology in their regular training programs within the care organization, and the data specialists who need to make decisions about incorporating the data provided by the digital care technology. Considering the increasing influence of digital care on several domains of the life of people with intellectual disabilities [13], the design and implementation of technologies should be well considered.

### Responsible Design and Implementation

Ideally, the design and implementation of digital care for people with intellectual disabilities are “responsible”: to include any intentional systematic effort designed to increase the likelihood of a digital care technology developed through ethical decision-making, being socially responsible and aligned with the values and well-being of those influenced by it [15]. Values, defined as “convictions or matters that people feel should be strived for in general and not just for themselves to be able to lead a good life or realise a good society” [16], are commonly considered within ethical discourse. These values function as moral compasses that guide certain actions, for example, in the design and implementation of technology. However, the ethics of digital care is not a common subject of study [17,18]. There is limited empirical evidence describing how to address stakeholders’ values within their context, in this case, the context of long-term care for people with intellectual disabilities, even though it is broadly recognized that responsible design and implementation of digital care require insight into and sensitivity toward specific contexts of use [17,19,20]. There is a need for context-specific studies about how certain values matter to the stakeholders of particular technologies and how these values can be accounted for in technology design and implementation.

### Guidance Ethics

The “guidance ethics approach” [21] is a relatively new method for reflection about and guidance for the responsible design and implementation of technologies in the context of use, developed by the ECP (Platform for the Information Society), the Netherlands. The approach is applied in various fields, such as municipalities, government, security, police, and health care sector, and regarding various cases, such as a biofeedback app for people with profound intellectual and multiple disabilities and challenging behavior or the use of artificial intelligence for nighttime monitoring in disability care [22]. The guidance ethics method involves a multistakeholder workshop, in which stakeholders’ values are identified based on an exploration of the experienced and expected positive and negative effects of using a specific technology. Subsequently, workshop participants mutually formulate action opportunities to account for these values in the specific context of use. In this study, we used this method to identify stakeholders’ values and formulate action opportunities for the responsible design and implementation of specific technologies used in long-term care for people with intellectual disabilities.

The guidance ethics approach—more extensively described in the *Methods* section—has several advantages compared with other research methods focused on ethics in design. One of the most well-known methodologies considering ethics through values in design is “Value Sensitive Design” [23]. Although the idea of embedding values in technology originated from this method, it does not provide the tools to empirically study values. However, guidance ethics is practical and hands-on, allowing stakeholders in a workshop to contribute to identifying the values affected by the use of technology. There are tools, such as the “interactive technology assessment” [24], that also provide hands-on tools, but these solely focus on studying the values of 1 user. Guidance ethics enables to involve a diverse group of stakeholders to identify a comprehensive set of values [7] and facilitates stakeholders to better understand the position of others [25]. Although there are methods, such as an evidence-informed, deliberative process approach to a health technology assessment [7,26] that involves multiple stakeholders also, this method, in contrary to guidance ethics, does not translate insights into concrete action opportunities for responsible technology use. To the best of our knowledge, guidance ethics is the only method that enables the study of values involving multiple stakeholders and directly translates these into concrete action opportunities.

### Objective

In this study, we applied the guidance ethics approach to three digital care technologies that are currently being developed or implemented within care organizations for people with intellectual disabilities:

1. The design of “Kookapp for groups” (developed by care organizations *Amerpoort* and *Reinaerde* and IT company *Ilionx*, Utrecht): this is a web application to support group workers with the preparation of healthy and tasty meals for groups of people with intellectual disabilities, from choosing recipes and buying ingredients to cooking and serving the meals.
2. The implementation of the “SignaLEREN” app (developed by care organization *Koraal* and IT company *Ivengi*, Maastricht): this app is used by people with intellectual disabilities or autism spectrum disorder to regularly gauge their emotional state; in the case of increased stress, they can choose a personalized stress-reducing activity within the app, such as watching a video clip or listening to certain music.
3. The implementation of SARA (developed by *SARA Robotics*, Eindhoven): this is a social robot that provides activities (eg, exercises, games, and music) during day care to stimulate interaction and physical activity among older people with intellectual disabilities.

With these 3 cases, we aimed to present examples of action opportunities to guide the responsible use of digital care for people with intellectual disabilities.

## Methods

### Participants

The 4 care organizations of the 3 cases participated in the Innovation Impulse Disability Care, a 3-year program initiated by the Dutch Ministry of Health, Welfare, and Sport in 2019. This program aimed to accelerate digital transformation in long-term care by providing support in implementing technology in the everyday practice of 26 disability care organizations [27]. In each organization, the implementation started by defining a topical care issue from the perspective of and together with people with a disability, followed by the selection of a technology that contributed to the solution of this care issue. In addition, organizations evaluated their IT and organizational readiness to implement the selected technology [28,29]. The care issues included, for example, improving day structure [30] or sleep-wake patterns [31], lowering stress levels, and increasing independent living [32]. Digital care technologies included sensors, domotics, social robotics, and apps. The Innovation Impulse program also entailed researching the factors influencing the implementation (NM Siebelink, unpublished data, 2024).

All 26 care organizations were invited to apply for participation in this study. Guidance ethics workshops were conducted in 4 care organizations, for the 3 cases described previously. Project leaders of the Innovation Impulse program within each care organization invited a purposefully diverse group of workshop participants from their organizations, for example, people with intellectual disabilities, relatives, care professionals, policy advisors, managers, members of the board of directors, IT staff, and technology developers.

In total, 26 individuals participated in this study. Participants’ characteristics are presented in Table 1. No personal data such as sex or age were collected. Almost all participants had some knowledge about the specific technology: of the 26 participants, 13 (50%) considered themselves informed, 9 (35%) had practical experience with the technology, and 4 (15%) were unfamiliar with the respective technology before participating in the workshop.

**Table 1:** Study participants’ characteristics.

Participant	Case 1: Kookapp for groups (n=10), n (%)	Case 2: SignaLEREN app (n=10), n (%)	Case 3: social robot, SARA (n=6), n (%)
People with intellectual disabilities (or a representative)	2 (20)	0 (0)	0 (0)
Management or policy maker	2 (20)	1 (10)	1 (17)
IT staff or technology developer	1 (10)	2 (20)	2 (33)
Care professional or team leader	4 (40)	3 (30)	2 (33)
Other (eg, project leader or consultant)	1 (10)	4 (40)	1 (17)

### Procedure and Materials

This study had a qualitative research design, using guidance ethics workshops to collect data. In total, three 3.5-hour multiple stakeholder workshops were conducted by trained workshop leaders from ECP (2 per workshop). The workshops were attended in person in May, June, and September 2022. Data were collected by means of a questionnaire (described in this section) completed on paper by the participants during the workshop. In addition, the information that the workshop leaders wrote on the flip charts was collected by taking photographs of the flip charts. In total, 2 researchers (KNvD and NMS or AvdP) were present during each workshop to observe and explain the study and the questionnaire; they did not engage in the workshop.

**Figure 1:**
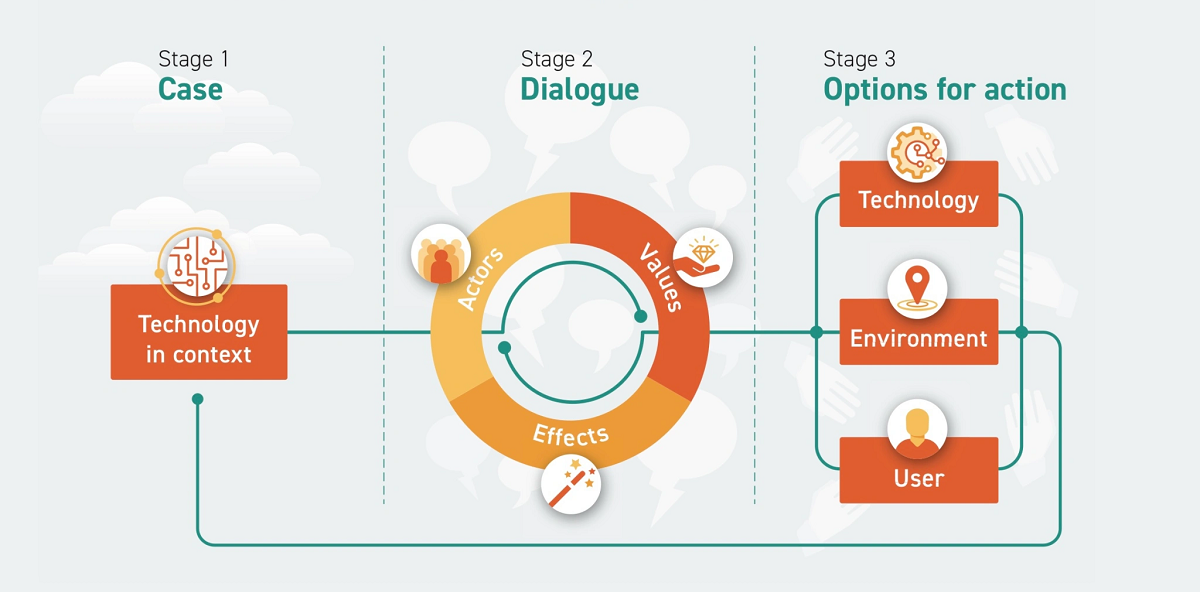
Outline of the guidance ethics approach (adapted from Verbeek and Tijink 2020 [21], which is published under Creative Commons Attribution 4.0 International License [33]).

The questionnaire—constructed by researchers (NMS and KNvD) for this study—followed the workshop outline (Figure 1 [21,33]). In stage 1 of the workshop (case), the project leader presented the case, that is, information about the technological solution, its aim, the way it works, for which target group, and in which daily (care) process. Thereafter, data about the participants’ characteristics and their familiarity with the technology were collected.

In stage 2 (dialogue), participants were first asked to call out all actors that are or should be affected by or involved with the use of the technology; the workshop leaders wrote these actors on a flip chart. Second, participants were asked to write down any positive and negative effects of the technology they could think of, not only from their own perspective but also any effect that came to mind. Next, all of them were asked to mention an effect until all effects were called out. Again, the workshop leader wrote these effects on a flip chart, and the effects were discussed, supplemented, and clustered by the workshop leaders and participants. Third, the workshop leaders identified values based on the clustered effects, and these values were adjusted in discussion with the participants. Finally, in stage 2, each participant determined the top 3 values that they deemed most important for their professional role in the particular case. These values were marked on the flip chart and discussed, after which the top 3 values of the total group were determined.

For stage 3 (action opportunities), participants were divided into 3 subgroups with diverse stakeholders in each subgroup. These subgroups were invited to come up with action opportunities to achieve a highly value-driven use of the respective technology in the context of the specific case, using the top 3 values of the group as starting point. A slight deviation from the protocol was that the group of the third case (social robot, SARA)—which was relatively small—was not divided into subgroups in stage 3 and did not explicitly focus on the top 3 values. The subgroups were instructed to come up with action opportunities for the following (respectively): the design of the technology (technology), shaping the environment or context of use (context), and adjusting the behavior of the users (users). Workshop leaders explained the meaning of “action opportunities” by using the example of the technology “car.” Driving a car should be safe (value); therefore, cars have seat belts and automatic brakes (action opportunities for the technology to improve safety), traffic lights and other infrastructure guide drivers (action opportunities for the environment to improve safety), and drivers practice driving and learn the rules before receiving a license (action opportunities for the user’s behavior to improve safety). Participants were asked to write down the action opportunities that came to mind, which were then discussed and collected on a flip chart in the subgroups. Each subgroup presented their action opportunities, which were discussed plenarily. At the end of the workshop, participants were asked to write down in the questionnaire any new effects, values, or action opportunities that came to mind that were not mentioned during the workshop.

### Ethical Considerations

Participants were informed about the study and privacy statement, and they provided consent by completing the questionnaire anonymously. The local Medical Research Ethics Committee Oost-Nederland deemed the research in the Innovation Impulse program not subject to the Medical Research Involving Human Subjects Act (“Wet medisch-wetenschappelijk onderzoek met mensen”; file 2021-8293).

### Analyses

Data from the questionnaires about positive and negative effects, values, and action opportunities were entered in Excel (Microsoft Corporation; 2018). The data set was checked for completeness using notes of the workshop observations, pictures of the flip charts from the workshops, and the workshop reports made by ECP’s workshop leaders. Analyses were conducted using a bottom-up approach: participants’ descriptions were the starting point leading to the derivation of themes. First, 2 researchers (KNvD and NMS) independently derived themes from the “effects data” per case. That is, effects regarding a similar subject were given a descriptive name. For example, “joyous end users” and “end users can experience more enjoyment” were named “enjoyment of end users,” which was then considered an effect theme. The 2 researchers compared and discussed their effect themes until consensus was reached. Next, the effect themes of all 3 cases were written on digital Post-it notes and visually arranged, so that related effect themes were near each other. Digital Post-it notes on which values were presented were added for each theme from which the values were abstracted. Furthermore, 2 researchers (KNvD and NMS) also derived themes from the action opportunity data per case. Next, analyses and discussions were conducted regarding which values were represented by the action opportunity themes. For example, the action opportunity theme “Give the person with intellectual disability some self-direction in the use of the technology” is mainly related to the value “autonomy,” whereas the action opportunity theme “keep the goal in mind and deploy technology as a means rather than a goal in itself” is related to the value “quality of care.” The preliminary results were presented and discussed in an interpretation meeting with workshop leaders and project leaders from the care organizations (participants from all 3 workshops were present). Input and feedback from this meeting were used for further analyses through an iterative process.

## Results

### Overview

The 3 workshops provided insight into how effects were translated into values and, subsequently, how values were translated into action opportunities for technology, context, and users of a specific technology. The numbers of collected effects, values, and action opportunities for each of the 3 cases are presented in Table 2. An overview of their content is provided in Multimedia Appendix 1; for readability, effects and action opportunities were shortened, and similar ones were combined in the overview.

Data about values and action opportunities were missing from a participant who could only attend the first part of the workshop. Furthermore, the person with intellectual disability from case 1 formulated effects and action opportunities together with a care professional.

**Table 2:** Number of participants per case and the amount of data collected.

Case	Participants (n=26), n (%)	Effects (n=232), n (%)	Values (n=33), n (%)	Action opportunities (n=156), n (%)
1—Kookapp for groups	10 (38.5)	102 (43.9)	13 (39.4)	74 (47.4)
2—SignaLEREN app	10 (38.5)	66 (28.4)	7 (21.2)	54 (34.6)
3—Social robot, SARA	6 (23.1)	64 (27.6)	13 (39.4)	28 (17.9)

### Examples of Action Opportunities

Table 3 presents examples of action opportunities for (1) designing “technology”; (2) shaping the “context” of use; and (3) adjusting the behavior of the “users” to guide the responsible use of digital care for people with intellectual disabilities. Given that describing all results (which can be found in Multimedia Appendix 1) is beyond the scope of this paper, Table 3 highlights 1 example per case based on one of the most prominent values in that case, and we have described the effects and action opportunities linked to that value. Following the examples, we have reflected about general observations within and overarching the 3 cases.

**Table 3:** Examples of combinations of effects, a value, and action opportunities for the 3 cases.

Cases and categories			Examples
**Case** **1: Kookapp for groups—a web application to support healthy cooking for groups of people with intellectual disabilities**			
	Selected value	Quality of care	
	Positive effects	Connectedness through choosing, cooking, and eating together Continuity of meal quality; not being dependent on care professionals’ skills Awareness of the importance of good nutrition More time for the people with intellectual disabilities Equality (differences regarding meals for people with intellectual disabilities between care organizations become small when they use the web application)	
	Negative effects	Excessive focus on health compared with enjoying tasty food	
	Action opportunities—technology	None mentioned regarding the selected value	
	Action opportunities—context (care organization)	Evaluate efficiency, health, and eating pleasure continuously Provide the care professionals with instructions about how to use the web application along with a manual	
	Action opportunities—users (care professionals)	Invest in understanding the web application to use it properly Have a backup plan for situations in which the web application does not work Know the dietary preferences and needs of each person with intellectual disability in the group Know what to do when a person with intellectual disability does not want to participate (or experiences less fun) in cooking with the web application	
**Case** **2** **: SignaLEREN app—an app** **to support people with intellectual disabilities in autonomously dealing with stress or anxiety**			
	Selected value	Autonomy	
	Positive effects	The following were the positive effects for the people with intellectual disabilities: More self-direction, independence, and personal autonomy An extra support option besides support from care professionals Increased awareness of own stress and its causes	
	Negative effects	Counterproductive effects of the app on stress if it does not work Less autonomy when using the app feels obligatory The app as a barrier to seeking contact with the care professional	
	Action opportunities—technology	Enable people with intellectual disabilities to make choices themselves: Set the regularity of question pop-ups in the app Disregard the notifications at unsuitable moments by choosing the response option “I don’t want to answer this question (right now)” Delete data Schedule an appointment with their care professional via the app	
	Action opportunities—context	Give people with intellectual disabilities access to the back end of the app, so that they can adjust specific content in the app themselves	
	Action opportunities—users	Give people with intellectual disabilities some self-direction regarding the use of the app: Discuss what using the app entails and what happens with the data Give guidance in setting up and using the app at their own pace Evaluate app use and effects frequently during coaching moments Support people with intellectual disabilities in a different way, eg, redirect them to the app first, “Have you completed the app?”	
**Case 3: Social robot, SARA—a robot to support the physical and social activities for people with intellectual disabilities**			
	Selected value	Privacy	
	Positive effects	None mentioned regarding the selected value	
	Negative effects	Risk of privacy infringement owing to storage and sharing of personal data Insufficient insight into what data are collected and stored when the functionalities of the robot are expanded	
	Action opportunities—technology	Differentiate between accounts for administrators and care professionals using the robot to restrict access to personal data Establish a maximum storage period for personal data before they are automatically deleted	
	Action opportunities—context	Train the care professionals using the robot in accordance with the privacy law Restrict the number of people who have access to personal data Read and reconsider the consent statements annually with people with intellectual disabilities (or their representatives) who use the robot—taking into account any changes in the functionalities of the robot and therefore storage of other data Revise the organization’s privacy policy When people with intellectual disabilities use the robot to have contact with relatives, the privacy of both should be protected: Use headphones during this contact Create a cozy private “corner” in the location	
	Action opportunities—users	None mentioned regarding the selected value	

### Observations About and Differences and Similarities Among the Cases

#### Effects

Part of the effects that were collected was case specific. For example, effects regarding healthy food were mentioned only in case 1 (Kookapp for groups), effects regarding insight into the stress of the person with intellectual disability were mentioned only in case 2 (SignaLEREN app), and effects regarding activation or development of cognitive skills of the person with intellectual disability were mentioned only in case 3 (social robot, SARA). Apart from case-specific effects, several general themes that were extracted from the effects across all 3 cases are presented in Table 4.

**Table 4:** General (ie, not case specific) effect themes, values, and action opportunity themes.

Categories and themes found across the 3 cases (Kookapp for groups; SignaLEREN app; and social robot, SARA)		Actors to whom the themes mainly apply
**Positive effects**		
	Customization of care	People with intellectual disabilities
	Increase in self-reliance or self-direction	People with intellectual disabilities
	Ease of work or job satisfaction	Care professionals
	Efficiency or labor saving or time saving	Care organizations
**Negative effects**		
	Dependency on IT infrastructure	People with intellectual disabilities, care professionals, and care organizations
	Risks related to the storage of privacy-sensitive data	People with intellectual disabilities, care professionals, and care organizations
	Perception of having “yet another system”	Care professionals
	Cost or time investment	Care organizations
**Values**		
	Quality of care	People with intellectual disabilities
	Autonomy	People with intellectual disabilities
	Privacy	People with intellectual disabilities, care professionals, and care organizations
	Job satisfaction	Care professionals
	Efficiency or affordability of care	Care organizations and society
**Action opportunities**		
	Keep the content (recipes, interventions, and activities) up to date	Care professionals
	Train the care professionals regarding how to use the technology well	Care organizations
	Connect the use of the technology to goals in the individual care plan or electronic health record	Care professionals and technology developers
	Focus on upscaling (more users of the technology in the care organization)	Care organizations

#### Values

Table 5 shows the values that were identified as the top 3 values during the 3 workshops. Note that only in case 1 (Kookapp for groups), participants chose 4 values. Values identified in all 3 cases were “quality of care,” “autonomy,” “privacy,” “job satisfaction,” and “efficiency or affordability of care” (Table 4). In all 3 cases, certain values apply to a specific actor. For example, “job satisfaction” applied to care professionals, whereas “autonomy” was primarily related to people with intellectual disabilities. Other values applied to several actors (eg, “reliability”) or an actor group; for example, “efficiency or affordability of care” was related to the care organization or even society.

**Table 5:** Top values identified during “guidance ethics” workshops, based on the personal top 3 values of all participants per case.

Case	Top values
1—Kookapp for groups	Quality of care, efficiency, health, and enjoyment
2—SignaLEREN app	Quality of care, autonomy, and reliability
3—Social robot, SARA	Quality of care, autonomy, and privacy

#### Action Opportunities

Action opportunities regarding the technology comprised the development of the technology itself (eg, optimization of user experience and customization), input into the technology (eg, recipes for meals in case 1—Kookapp for groups; intervention options for reducing tension in case 2—SignaLEREN app; and activities in case 3—social robot, SARA), and output of the technology (eg, storage and use of data). Action opportunities regarding the context covered the need for guidelines, training and support, policy or agreements, and adjustments to the physical environment in which the technology is used. Action opportunities regarding the users mainly focused on how care professionals can integrate the technology into their daily care practice. Some behaviors need to be diminished (eg, care professionals need to “let go” instead of “take over” to give the person with intellectual disability more autonomy), some behaviors must be retained (eg, face-to-face contact moments), and some behaviors need to be added (eg, evaluation moments). Although some general themes across all 3 cases were identified (Table 4), most action opportunities were context specific.

For case 1 (Kookapp for groups), most action opportunities were listed for the value “user convenience.” Action opportunities included optimizing the user experience with the web application (eg, using icons and less text) and providing resources (eg, placing magnets on the kitchen wall to hold tablets while cooking). Action opportunities that stood out because they were mentioned by several participants were related to an attractive design and ease of operation of the web application. Although the value “health” was a top value in case 1, relatively few action opportunities were formulated for this value.

For case 2 (SignaLEREN app), none of the values stood out, but 4 values were evenly represented among most action opportunities: “quality of care,” “autonomy” (Table 3), “reliability,” and “efficiency.” “Quality of care,” “autonomy,” and “reliability” were the top 3 values. For “quality of care,” action opportunities included keeping the goal in mind and deploying the app as a means rather than a goal in itself (eg, personal goals of the person with intellectual disability as starting point for the conversation about how to use the app), training the care professionals on the use of the app, and having a person-centered approach (ie, customizing the app). Action opportunities for the value “reliability” included continuous provision of easily accessible support (eg, assigning SignaLEREN coaches and arranging a 24-h helpdesk) and the maintenance of the app organized within the own organization. Finally, action opportunities for the value “efficiency” included integrating the app in the care process (eg, embedding the use of the app in a particular care methodology), scaling up the use and adoption of the app (eg, deploying the app with all care professionals to whom it applies), and extracting and using data from the app (eg, built-in notifications in the app for when the person’s stress level is likely to become very high). The more frequently mentioned action opportunities included giving the person with intellectual disability access to the personal settings of the app (eg, frequency of prompts and data access rights), securing face-to-face contact of the person with intellectual disability and care professional, using data from the app to provide insights into stress level trends, connecting the app with the electronic health record, and assigning a SignaLEREN coach.

For case 3 (social robot, SARA), most action opportunities were related to the value “privacy” (Table 3). Action opportunities that stood out because they were mentioned by several participants included linking the use of the robot to individual care goals, connecting the robot with the electronic health record, and expanding the content that the robot can present. Although the value “autonomy” was a top value in case 3, relatively few action opportunities were related to this value, whereas relatively many action opportunities were linked to “effectiveness” (eg, optimizing the content that the robot can present and recurrent evaluation), which was not chosen as a top value.

## Discussion

### Relevance

Often, the development, implementation, and use of digital care does not entail an intentional and systematic effort to include the ethical considerations of all involved stakeholders [34]. Therefore, new technologies and the processes to integrate them into daily practice are often not aligned with the values and well-being of those influenced by them [15]. Instead, ethics is merely considered a separate area of attention (eg, a separate line of investigation or work package within projects) discussed by a distinct group of experts [35].

This study illustrates the types of insights that are gained when various stakeholders are involved in the reflection about the ethical impact of specific technologies and how this impact can be influenced for the better. This is illustrated using 3 cases of different digital care technologies for people with intellectual disabilities: a web application for cooking for groups (Kookapp for groups), an app for stress regulation (SignaLEREN app), and a robot for interaction and physical activity (social robot, SARA). Our findings may help researchers, innovators, and users of technology to move from a rather abstract thinking about ethics and responsible innovation toward effective practical approaches in which all stakeholders can be involved.

### Principal Findings

In a short amount of time (three 3.5-h workshops), relatively much information was gathered in a multistakeholder setting regarding (1) positive and negative effects for various stakeholders of a specific digital care technology for people with intellectual disabilities; (2) values underlying these effects; and (3) action opportunities to take into account important values in the design, implementation, and use of the specific technology. The effects were primarily case specific, as they described the implementation of a technology in a specific context, but several general themes were also recognized. The latter included the effects of the technology on customization of care, dependency on IT infrastructure, self-reliance or self-direction of people with intellectual disabilities, risk of privacy infringement, care professionals’ ease of work, workload, and efficiency and investment. When all the effects were abstracted into values, several values were identified in all 3 cases and were found to be related to the general effects. These values were quality of care, autonomy, privacy, job satisfaction, and efficiency or affordability of care. Most action opportunities were related to the top values from the respective cases, as can be expected when the guidance ethics approach (stage 3) is followed. Hence, many action opportunities from case 1 (Kookapp for groups) were related to “enjoyment,” “efficiency,” and “quality of care.” However, relatively few action opportunities involved the top value, “health.” Notably, most action opportunities from case 1 were related to “user convenience”; however, this was not identified as a top value. In case 2 (SignaLEREN app), most action opportunities were related to the top values (“quality of care,” “autonomy,” and “reliability”) and to “efficiency.” In case 3 (social robot, SARA), the top values, “quality of care” and “privacy,” were well represented among the action opportunities, but few were related to the top value, “autonomy,” and relatively many were related to “efficiency” and “effectivity.”

Although action opportunities can only be described in relation to specific sociomaterial contexts [36] (ie, specific technologies in their contexts of use), our study reveals that, at a higher level, there are similarities regarding effects, values, and action opportunities for different cases. Thus, it may be wise for stakeholders of digital care technologies to not only learn how technologies can be responsibly used within their own context but also seek inspiration from similar contexts in which other technologies are used and from different contexts in which the same or comparable technologies are used. However, caution should be exercised when generalizing case-specific effects, values, and action opportunities to a broad scope.

### Comparisons With Previous Studies

This is the first study to provide a broad overview of actual action opportunities for responsible digital care for people with intellectual disabilities. In their 2020 reports, the Dutch Centre of Ethics and Health advised the Dutch Ministry of Health, Welfare, and Sport about ethics regarding digital care such as apps and robots [37,38]. The themes discussed in the report regarding apps are cost savings, increase of autonomy, increase of well-being, unrest, information overload, decrease of human contact, overemphasis on health that can lead to medicalization, and increase of differences in health and inequality [37]. Notably, the diametrically opposed side of inequality was raised in our study, namely that differences between care organizations would decrease if they would organize meals using the Kookapp for groups. The report regarding care robots discusses meaningful contact, dignity, autonomy, dependency, privacy, and justice [38]. Apart from information overload and justice, all themes also appeared in ≥1 of the 3 cases in our study. This shows that our results covered most of the essential topics that ethics experts recognized.

Studies of ethics and health often describe themes that have a positive or negative load (eg, increase of autonomy or increase of inequality, respectively) or identify ethical harms [17,18,37,39]. Our data revealed that, in most cases, 2 sides of the same coin were considered in the multistakeholder setting, for instance, technology as a facilitator and a burden for care professionals’ work (all cases); cost or labor savings and high costs or time investment (all cases); positive and negative effects of the focus on a health theme (Kookapp for groups: awareness of the importance of healthy food vs a lot of emphasis on health at the cost of enjoying tasty food; SignaLEREN app: improving the stress signaling plan vs risk of medicalization of normal stress); and increase and risks of autonomy (SignaLEREN app: person with intellectual disability is less dependent on care professional but possibly also less “visible”). The advantage of using values—instead of themes or harms—as a starting point for fostering responsible use of digital care is that values are neutral and hence facilitate the consideration of both sides of the same coin [40].

### Strengths and Limitations

As this study illustrates, the guidance ethics approach can be a valuable and low-key method to gain insight into different stakeholders’ experienced and expected positive and negative effects and values affected (or at stake) when using a specific technology and insight into action opportunities for responsible digital care. However, we recognize that the insights gained in this respect may fall short in terms of correctness (ie, being in agreement with facts or with what is generally accepted), concreteness (ie, being specific and detailed), and completeness (ie, the extent to which all relevant effects, values, and action opportunities have been identified) [41].

The correctness of our results about effects may be limited owing to, among others, a general lack of methodologically sound studies of the effects of digital care for people with intellectual disabilities. Hence, there was little to no evidence from scientific studies of the specific care technologies to be presented in stage 1 of the workshops. Therefore, the collected positive and negative effects are mainly based on subjective effects but from stakeholders with lived experience with the specific technology. In addition, an inherent characteristic of qualitative data analysis is that deriving themes from the data (including identifying values based on the effects of the technology) involves interpretation by the analyst. To limit subjectivity, values were discussed during the workshop with all participants, and themes were created independently by 2 researchers and discussed until consensus was reached. Another discussion point linked to correctness is that the values were discussed and presented as relatively stable entities, while they are neither stable nor singular [42,43]. Values may be affected by time and thus constantly defined and redefined (value dynamism), for instance, because users have gained experience with technology [41,43]. Although it may be challenging for researchers, innovators, and other stakeholders to continuously respond to this dynamism, a starting point could be to regularly collect the stakeholders’ perspectives about effects, values, and action opportunities.

The method used in this study has advantages regarding concreteness. For example, although the values are abstract, their definitions are embedded in the concrete effects from which they are derived. Moreover, the method results in relatively concrete output (action opportunities) compared with most ethics research on digital care [44,45], and action opportunities apply to the specific context of use. However, it was not always deducible from the workshop data what a participant specifically meant by an effect or action opportunity or to whom (eg, care professional or person with intellectual disability) specific insights applied. In the cases used in this study, it is not straightforward who is meant by the “user” of the care technology. To improve this, workshop leaders should be alert and ask participants to clarify whether they mean the care professional or the person with intellectual disability.

Regarding completeness, this study did not aim to be exhaustive in collecting effects, values, and action opportunities. However, we aimed to include a diverse sample of participants. Despite the accessibility of the workshops for people with intellectual disabilities, a participant with intellectual disability was included only in case 1 (Kookapp for groups). In case 3 (social robot, SARA), the person with intellectual disability withdrew on the morning of the workshop (with a valid reason), and in case 2 (SignaLEREN app), no person with intellectual disability was invited. In addition, other relevant stakeholders were absent, for example, relatives of the people with intellectual disabilities, the board of directors, or representatives of health insurers. Although all relevant stakeholders that participants could think of were identified during the workshop and participants were asked to keep them all in mind, some perspectives may be missing in the results. Including people with disabilities requires special attention, as this is not common in the co-design or cocreation of digital care technologies [46]; however, this is upcoming [13,47,48]. To improve stakeholder inclusion in general, it may be useful to consult “design principles” for stakeholder engagement [49].

Furthermore, the method of deriving values from effects is suitable for identifying “proximal” values on the micro level that are specific to the technology and context of use [21], but mesolevel or macrolevel effects and more “distal” values may be missed [50] (such as “social justice,” which is an important theme in studies of ethics and digital care [45,51,52]). Whenever missed in bottom-up ethical dialogues (such as in this study), proximal and distal values (or principles) from predefined ethical frameworks could be brought in as “top-down” guidance. At the same time, the bottom-up approach is a strength of the guidance ethics approach, revealing important topics such as enjoyment of the person with intellectual disability or job satisfaction, which may be missed when an ethical theory is applied to a case instead [17,53]. In this sense, we argue that the top-down and bottom-up approaches are complementary. Hence, we suggest moving back and forth between the perspectives of stakeholders affected by technology when implementing digital care and ethical frameworks or perspectives of experts about digital care ethics [19,54].

Finally, the action opportunities identified in this study require follow-up in practice. Responsible use of technology requires being continuously responsive and adaptive to new insights that are gained regarding effects, values, and action opportunities, from early design to local implementation and use [55,56]. In addition, it is conceivable that trade-offs between action opportunities need to be made owing to value conflicts (eg, autonomy vs duty of care [57]) and costs. Future studies may shed light on how action opportunities, once formulated, are further operationalized and applied by technology designers, user organizations, and individual end users of the technology and on what factors withhold stakeholders from doing so. Previous studies indicated that ethical concerns of stakeholders might considerably slow the pace of digital care innovation, implying that responsible innovation could be a core catalyst for the progress of digital care overall [18]. Through explicit attention to and communication about responsible digital care, not only are ethical concerns taken into account but also support and acceptance among the involved stakeholders are generated. This increases the chances for the successful implementation of socially desirable, ethically acceptable, and sustainable digital care that improves the lives of people with disabilities.

## Appendix

Multimedia Appendix 1 Overview of the effects, values, and action opportunities collected from the workshops for the 3 cases: Kookapp for groups; SignaLEREN app; and social robot, SARA.

## Abbreviations

ECP: Platform for the Information Society
