# Supplementary material for: Action Opportunities to Pursue Responsible Digital Care for People With Intellectual Disabilities: Qualitative Study
Source: JMIR Ment Health. 2024 Feb 28;11:e48147. doi: 10.2196/48147 (PMC10938230; doi:10.2196/48147)
Supplement: Multimedia Appendix 1 [file mental_v11i1e48147_app1.docx]

# Multimedia Appendix 1

Appendix to:

Siebelink, N.; van Dam, K.; Lukkien, D.; Boon, B.; Smits, M.; van der Poel, A. Action opportunities to Pursue Responsible Digital Care for Persons With Intellectual Disabilities: Qualitative Study *JMIR Mental Health*

Overviews of effects, values, and action opportunities collected in the workshops for the three cases: Kookapp for groups, SignaLEREN app, social robot SARA.

| Case 1 \| Kookapp for groups; a web app to support healthy cooking for groups of persons with ID | | |
| --- | --- | --- |
| **(Possible) Effects** | **Values** | **Action opportunities for the three domains** |
|  | ^a^ top values |  |
| *Positive* | Efficiency ^a^ | *Technology* |
| - Easier cooking process; more structure and overview |  | - Make the web app easy to use for persons with ID and care professionals with less digital skills by limiting text and clicks, no login, emojis and a reading functionality |
|  | Health ^a^ |  |
| - More efficient cooking process; better use of time |  |  |
|  | Quality of care ^a^ |  |
| - Persons with ID feel more involved in deciding over what they are eating and cooking |  | - Make sure the web app runs on diverse operating systems |
|  | Enjoyment ^a^ |  |
|  |  | - Make the web app appealing with pictures, colours, themes, and the possibility of earning points |
| - Increased choice for persons with ID | User convenience |  |
| - Connectedness through choosing, cooking, and eating together |  |  |
|  |  | - Indicate the ‘health score’ of recipes with emojis and number of calories, and highlight healthy recipes |
| - More time for the person with ID | Sustainability |  |
| - More fun with cooking and eating |  |  |
| - Persons with ID and their care professionals feeling better | Inclusiveness | - Provide a feedback function to ask how users liked the food |
|  |  |  |
| - Varied and healthy meals | Privacy | - Add the possibility to repeat meals and schedule these as repeat days |
| - Better portion sizes (portion control) |  |  |
| - Continuity of meal quality, independent of care professionals’ skills | Autonomy |  |
|  |  | *Context* |
| - Awareness of the importance of good nutrition | Job satisfaction | - Provide care professionals with instructions on how to use the web app, also in a manual |
| - Increased sustainability and less waste, because leftovers are used the next day | (Technological) robustness | - Include colleagues who are less enthusiastic and discuss their opinions and objections |
| - Accessible everywhere |  | - As managers, instruct and motivate care professionals to use the web app |
| - Better management of finances; reduction of costs | Support |  |
|  |  | - Manage the use of the web app as care organisation |
| - Improved compatibility with electronic health record | Control/ manageability |  |
|  |  | - Create awareness that the web app saves preparation time |
| - Positive image as care organisation, because persons with ID also want to cook there |  |  |
|  |  | - Create awareness about the importance of healthy food |
| - More volunteers motivated to cook, because the web app helps to make cooking easier for them |  | - Formulate a vision on healthy living as care organisation |
|  |  | - Ensure that the web app complies with personal data laws (General Data Protection Regulation) |
| - Being distinctive as care organisation |  |  |
| - More collaboration between care organisations involved in the development and maintenance of the web app |  |  |
|  |  | - Provide insight into current time use and actions regarding the cooking process (without the web app) |
| - Equality (differences regarding meals for persons with ID between care organizations become smaller when they use the web app) |  |  |
|  |  | - Measure efficiency, health, and eating pleasure continuously |
|  |  | - Keep track of changes in dietary preferences of persons with ID |
| *Negative* |  | - Guarantee sufficient time around the cooking process |
| - Not enough customization for specific dietary preferences and needs |  |  |
|  |  | - Create a cosy dining area |
| - Excessive focus on health compared to enjoying tasty food |  | - Provide kitchen wall magnets for tablets to be used while cooking |
|  |  |  |
| - Restricted view in recipes and dependence on product availability of ingredients; less room for creativity of the chef |  | - Provide suitable devices and stable Wi-Fi connection |
|  |  |  |
|  |  | - Set up a helpdesk, eventually via an agreement with the technology supplier |
|  |  |  |
| - Persons with ID and/or care professionals experiencing less autonomy |  | - Make agreements about prices and possible discounts with the food supplier |
|  |  |  |
|  |  |  |
| - Dissatisfaction if ‘your’ recipe is not listed in the web app |  | *User* |
|  |  | - Ask people with ID for feedback |
| - Disagreement when persons with ID cannot agree on the menu |  | - Involve and motivate persons with ID, make them a part of the recipe selection and cooking process |
|  |  |  |
| - Fallback into old patterns when the interest wanes |  |  |
|  |  | - Know what to do when a person with ID does not want to participate (or experiences less fun) in cooking with the web app |
| - Having to work with a new system can demotivate care professionals |  |  |
|  |  |  |
| - Costs for finetuning and customisation of the web app, especially in case of technical complexity due to specific dietary preferences or needs |  | - Facilitate shared decision-making in choosing recipes |
|  |  |  |
|  |  | - Make nutrition a fun and important theme in the groups |
|  |  |  |
| - Costs and time for implementation |  | - Know the dietary preferences and needs of each person with ID in the group |
| - Potentially higher costs for ingredients |  |  |
| - Need for more devices and IT support |  | - Invest in getting to know the web app in order to use it properly |
| - Possibly not compatible with every electronic health record |  |  |
|  |  | - Align the use of the web app with targets set in the personal care plan of each person with ID |
| - Risk of privacy infringement |  |  |
| - Increased dependency on technology; causing issues in case of e.g. Wi-Fi failure or device change |  |  |
|  |  | - Focus on variation when choosing recipes for a weekly menu |
|  |  |  |
|  |  | - Do not deviate (too much) from the recipe |
|  |  | - Have a back-up plan for situations in which the web app does not work |
|  |  |  |
|  |  | - Report hiccups to improve the web app |

| Case 2 \| SignaLEREN app; an app to support people with ID in autonomously dealing with stress | | |
| --- | --- | --- |
| **(Possible) Effects** | **Values** | **Action opportunities for the three domains** |
|  | ^a^ top values |  |
| *Positive* | Quality of care ^a^ | *Technology* |
| - More self-direction, independency and autonomy |  | - Make the app user-friendly (e.g. voice control, icons) |
|  | Autonomy ^a^ |  |
| - An extra support option besides support from care professionals |  | - Add the answer option ‘I don’t want to answer this question (now)’ |
|  | Reliability ^a^ |  |
| - More calmness for the person with ID and their loved ones |  | - Enable persons with ID to set the regularity of question pop-ups (prompts) in the app |
|  | Job satisfaction |  |
| - Persons with ID have an increased awareness of their stress and its causes |  | - Enable persons with ID to delete certain data in the app |
|  | Efficiency |  |
| - Concrete input and more in-depth coaching moments for the person with ID with the care professional |  | - Add a help function for persons with ID in the app |
|  | Involvement |  |
|  |  | - Link the app to agenda so that the person with ID can schedule an appointment with their care professional |
| - More focus on the individual care plan of the person with ID | Privacy |  |
|  |  |  |
| - Insight into the stress level of the person with ID over a period of time; better understanding of the well-being of the person with ID |  | - Link the app to relatives of the person with ID with pop-up notifications of what they can do in cases of high stress levels in the person with ID |
|  |  |  |
|  |  |  |
|  |  |  |
| - Early identification of issues; patterns can be discovered more easily and causes are easier to trace |  | - Set thresholds in the system to gain insight into the stress level of the person with ID |
|  |  |  |
|  |  | - Compose a graph from data that shows patterns of the stress level of the person with ID |
| - Support for the person with ID is faster and more efficient |  |  |
|  |  |  |
| - Reduced workload for care professionals |  | - Link the app to the electronic health record to gain insight into patterns of stress of the person with ID |
|  |  |  |
| - More time available to respond to other care requests from (other) persons with ID |  |  |
|  |  | - Make the app widely available to be used on many devices and operating systems |
|  |  |  |
| - Easier transmission in case of a change of caregivers; key insights and learnings about needs of persons with ID can be retrieved through the app |  |  |
|  |  | *Context* |
|  |  | - Give the person with ID access to the back-office |
|  |  |  |
|  |  | - Provide a telephone number for ICT helpdesk, which is 24/7 available for support |
| *Negative* |  |  |
| - Counterproductive effects of the app on stress if it does not work (e.g. due to system failure) |  | - Appoint a coach for each person with ID and their care professional using the app |
|  |  |  |
|  |  | - Train employees in using the app |
| - Risk of privacy infringement |  | - Integrate the app into the care process and establish as a systematic way of working |
| - Multitude of systems |  |  |
| - Registration burden for care professionals |  | - Maintain agreements regarding contact between persons with ID and their care professionals |
|  |  |  |
| - Not all care professionals can (equally) participate in technology use due to limited digital literacy |  |  |
|  |  | - Record agreements on how to deal with outcomes |
|  |  |  |
| - Starting the use of the app with the person with ID takes a lot of time |  | - Add a periodic reminder in the electronic health record to update the care plan of the person with ID based on insights gained through the app |
|  |  |  |
| - Disappointment if after a considerable investment of time and effort it turns out that the app is not a suitable solution for the specific person with ID |  |  |
|  |  |  |
|  |  | - Educate employees about stress and stress management |
|  |  |  |
| - The person with ID must always carry their smartphone |  | - Establish a user group to collect new ideas |
|  |  | - Determine who has access to which data |
| - Less autonomy when using the app feels obligatory |  | - Organise functional management of the app within the care organisation |
|  |  |  |
| - Decrease in conversational skills of the person with ID and their care professional |  | - Provide smartphones to persons with ID as care organisation |
|  |  |  |
|  |  |  |
| - Lack of personal contact; fewer spontaneous interactions between the person with ID and their care professional |  | *User* |
|  |  | - Provide instructions on correct use of the app |
|  |  |  |
|  |  | - Discuss with the person with ID what the use of the app entails and what happens with the data |
| - Persons with ID could experience the app as a barrier in seeking contact with the care professional |  |  |
|  |  |  |
|  |  | - Discuss in advance for what purpose the app will be used and which contact moments will be kept |
| - Possible reduced view of the wellbeing and process of the person with ID (in case of less contact with the care professional) |  |  |
|  |  |  |
|  |  | - Guide the person with ID in setting up and using the app at their own pace |
|  |  |  |
| - More self-direction for the person with ID can entail risks (e.g. escalation when the app does not support in dealing with stress) |  | - Evaluate the use and effects of the app frequently during coaching moments |
|  |  |  |
|  |  | - As care professional, go through the data at least once a week |
|  |  |  |
|  |  | - Keep in touch face-to-face, regardless of the insights gained through the app |
|  |  |  |

| Case 3 \| Social robot SARA; a robot to support physical and social activities for persons with ID | | |
| --- | --- | --- |
| **(Possible) Effects** | **Values** | **Action opportunities for the three domains** |
|  | ^a^ top values |  |
| *Positive* | Quality of care ^a^ | *Technology* |
| - Physical and cognitive progress, activation of the person with ID |  | - Make the programs on the robot easier to use for the person with ID |
|  | Autonomy ^a^ |  |
| - More variety and entertainment in the activity programs offered at the day care |  | - Offer more (diversity in) content for the disability care sector |
|  | Privacy ^a^ |  |
|  |  | - Enable putting in tasks related to activities of daily living tasks and daily structure in the robot |
| - Non-judgemental, neutral approach of the robot in all situations (compared to care professionals) | Effectiveness |  |
|  |  |  |
|  | Equality | - Enable self-learning ability for the robot |
| - More self-reliance for the person with ID |  | - Make a distinction between administrator accounts and accounts for individual care professionals in using the robot, in order to restrict access to personal data |
|  | Humanness |  |
| - More attention and proximity to the person with ID; less loneliness |  |  |
|  | Job satisfaction |  |
| - Relatives may be more actively involved (via the robot) |  | - Link with other systems |
|  | Collaboration | - Establish a maximum data storage period for personal data, before it is automatically deleted |
| - Reduction of workload for employees |  |  |
| - The robot can be part of the work process | Image |  |
|  |  |  |
| - Reduction of costs | Affordability of care | *Context* |
| - Improved image of the organisation; progressive |  | - Create a cosy private ‘corner’ for contact with relatives |
|  |  |  |
| - Attractive PR to recruit new employees and persons with ID | Wellbeing | - Train care professionals using the robot in accordance with the privacy law |
|  |  |  |
|  | Health | - Restrict the number of persons who have access to personal data |
| *Negative* |  |  |
| - Boring routine if activities are not renewed occasionally | Control/ manageability | - Complete the current care organisation’s policy on privacy |
| - Resistance of relatives |  | - Review, supplement and/or discuss consent statements yearly with persons with ID (or their representatives) who use the robot taking into account any changes in functionalities of the robot and therefore storage of other data |
| - Robots are experienced as a replacement by some employees |  |  |
|  |  |  |
| - Dependence on technology; what if there is a malfunction? |  |  |
|  |  |  |
| - Human judgment may disappear when decisions are automatically made based on data (e.g. by intervening prior to escalation based on sensor data) |  |  |
|  |  | - Put further development on the agenda of the care organisation once per quarter |
|  |  |  |
|  |  |  |
| - Dehumanization of care; ‘colder care’, depersonalisation |  | *User* |
|  |  | - Use headphones when the person with ID uses the robot for contact with relatives, in order to ensure privacy |
| - Different systems for care professionals |  |  |
| - Costs |  | - Deploy the robot individually with all persons with ID in a group |
| - Risk of privacy infringement due to storage and sharing of personal data |  |  |
|  |  | - Deploy the robot with an appropriate program to support a specific goal of an individual person with ID |
| - Insufficient insight into what data are collected and stored when functionalities of the robot are expanded |  |  |
|  |  |  |
|  |  | - Put in program components, reminders, and/or assignments that are linked to planning (for example: “Just 5 more minutes before we start wrapping up for today, you can start tidying up”) |
|  |  |  |
|  |  |  |
|  |  |  |
|  |  |  |
